# A Rare Etiology of 46,XY Disorder of Sex Development and Adrenal Insufficiency: A Case of MIRAGE Syndrome Caused by Mutations in the *SAMD9* Gene

**DOI:** 10.4274/jcrpe.galenos.2019.2019.0053

**Published:** 2020-06-03

**Authors:** Eda Mengen, Aynur Küçükçongar Yavaş, S. Ahmet Uçaktürk

**Affiliations:** 1 Ankara City Hospital, Children’s Hospital, Clinic of Pediatric Endocrinology, Ankara, Turkey; 2 Ankara City Hospital, Children’s Hospital, Clinic of Pediatric Metabolism, Ankara, Turkey

**Keywords:** Adrenal hypoplasia, 46,XY disorder of sex development, MIRAGE syndrome

## Abstract

Adrenal hypoplasia is a rare congenital disorder. In spite of biochemical and molecular genetic evaluation, etiology in many patients with adrenal hypoplasia is not clear. MIRAGE syndrome is a recently recognized congenital disorder characterized by myelodysplasia, infection, growth restriction, adrenal hypoplasia, genital phenotypes, and enteropathy. Here we present a case of MIRAGE syndrome due to a heterozygous missense variant (c.2920G>A; p.E974K) mutation in the sterile alpha motif domain-containing protein-9 (*SAMD9*) gene. This report describes the first MIRAGE syndrome patient in Turkey.

**What is already known on this topic?**

The recently described MIRAGE syndrome, which has autosomal dominant inheritance, is a very rare form of syndromic adrenal hypoplasia.

**What this study adds?**

Here, we presented the first syndromic adrenal hypoplasia case which was diagnosed with MIRAGE syndrome in Turkey.

## Introduction

Normal gonadal differentiation and sex development depend on the meticulous choreography and synchrony of a network of endocrine, paracrine, and autocrine signaling pathways, reflecting the actions and interactions of specific genes, transcription factors and hormones. Perturbations of this intricate network of gene regulation and gene expression governing fetal gonadal development result in disorders of sex development (DSD). These disorders are congenital and involve a spectrum of abnormalities in which the chromosomal, genetic, gonadal, hormonal or anatomical aspects of the sex are atypical (1). DSD patients have been grouped according to karyotype: 46,XY DSD, 46,XX DSD and sex chromosome DSD (1). However, due to the complexities of chromosomal and gonadal development, some diagnoses can be included in more than one of the three major categories. The number of genes identified as being involved in sex development continues to increase. Nevertheless, despite many recent genetic advances, the specific molecular etiology of the genital ambiguity in an individual cannot always be identified.

The recently described MIRAGE syndrome (OMIM# 617053), which has autosomal dominant inheritance, is a very rare form of syndromic adrenal hypoplasia. Its prevalence is <1/1000000 and the six core characteristic features are myelodysplasia, recurrent invasive infections, growth restriction, adrenal hypoplasia, genitalia anomalies, and enteropathy. Additional associated features are variable and include prematurity, chronic lung disease, developmental delay, dysmorphism and central nervous system anomalies (2,3).

The cause of the syndrome is germ line, heterozygous *SAMD9* variants that usually occur de novo leading to gain of function mutations in *SAMD9*. The cytogenetic location is 7q21.2, which is the long (q) arm of chromosome 7 at position 21.2. This gene encodes a sterile alpha motif domain-containing protein and is widely expressed (expressed in 208 organs). The protein product localizes to the cytoplasm and may play a role in regulating cell proliferation and apoptosis (2,3). *SAMD9* is likely to act as a growth repressor expressed in endothelial cells, and to lesser degree in fibroblasts. Pathogenic variants in the *SAMD9* gene consequently result in excessive growth-restricting activity intrinsic to the protein (4).

We present here a case of MIRAGE syndrome due to a heterozygous missense variant (c.2920G>A; p.E974K) mutation in the *SAMD9* gene.

## Case Report

The patient who was five months old presented to our hospital with fever, lack of oral intake, vomiting, and diarrhea. Due to a diagnosis of adrenal insufficiency, the patient was referred to Pediatric Endocrinology Unit after being managed by the inpatient clinic of the infectious diseases department. The medical history revealed that the patient, the third live-born among five pregnancies of a healthy 32-year-old mother, was prematurely born by cesarean section in the 31^st^ gestational week with a birth weight of 930 grams (<3^rd^ percentile) with severe intrauterine growth retardation. The parents were nonconsanguineous and there was no family history of adrenal insufficiency. It was reported that the patient required resuscitation followed by endotracheal intubation due to postnatal respiratory difficulty and was treated with surfactant due to respiratory distress syndrome. In addition the patient medical history included mechanical ventilation for six weeks and intravenous immunoglobulin (IVIG) treatment, which was started for thrombocytopenia that was detected during the follow-up. Steroid therapy and oral salt supplementation were started after the patient was diagnosed with adrenal insufficiency after skin hyperpigmentation was observed on the fifteenth postnatal day. Table 1 shows the results of hormone profile evaluation at 15 days of age and at five months of age. The patient was discharged from an external medical center after four months of treatment in the intensive care unit. At the age five months old, the initial physical examination showed a weight of 3850 grams (<3^rd^ percentile), height of 57 cm (<3^rd^ percentile) and a head circumference of 36 cm (3^rd^ percentile). The patient could not support the head and frontal bossing was present. External genital examination showed a pubic hair Tanner stage 1 with testes 1 ml bilaterally, in the middle and proximal portions of the inguinal canal. His stretched penile length was 2.0 cm. The patient was reported to receive 30 mg/m^2^/day hydrocortisone therapy. During follow-up, diarrhea was resistant to therapy and there was enteropathy that indicated colitis. The patient also had thrombocytopenia and/or anemia that required recurrent transfusions. IVIG therapy for thrombocytopenia was again administered. As the patient could not tolerate oral intake, nutritional support was provided by nasogastric tube feeding. For respiratory distress, intermittent oxygen was supplied by nasal cannula. Combined antibiotic treatment was applied for recurrent attacks of infection and sepsis. A stress dose of IV hydrocortisone (100 mg/m^2^/day) was administered. Serum electrolytes were normal. Therefore, fludrocortisone acetate and sodium chloride were not administered. Table 1 presents the results of laboratory tests for adrenal insufficiency and DSD in the patient. Scrotal ultrasonography imaging; the right testis, which was measured as 9x8x4.5 mm (vol: 0.2 cc), was localized in the middle portion of the right inguinal canal, while the left testis, which was measured as 6x8x8 mm (vol: 0.2 cc), was observed in the proximal portion of the left inguinal canal. Surrenal ultrasonography imaging; there was no imaging evidence that indicated the presence of adrenal glands, so bilateral adrenal hypoplasia was assumed. The peripheral chromosomal analysis resulted in a 46,XY karyotype. The presence of adrenal insufficiency, adrenal hypoplasia, the anomaly of the genitalia, resistant diarrhea, invasive infections and recurrent thrombocytopenia with episodes of anemia were compatible with MIRAGE syndrome. The results of bone marrow aspiration biopsy for delineating the etiology of thrombocytopenia, anemia and neutropenia were not compatible with myelodysplastic syndrome (MDS). The cytogenetic investigation of bone marrow revealed a 45,XY, -7 [4]/46,XY [3] karyotype. This result was described as mosaic monosomy 7. Therefore, SAMD9 sequencing was performed and identified a heterozygous missense variant c.2920G>A; p.E974K mutation in exon 3 in the SAMD9 gene. As the patient developed intolerance to oral intake due to vomiting and diarrhea, the decision to cease enteral feeding and to start total parenteral nutrition was made. The patient was admitted to the intensive care unit upon worsening of the general condition with tachypnea, tachycardia, and fever. Hypotension, decrease in respiratory sounds and coagulopathy developed during the follow-up and the patient was lost due to multisystem organ failure.

## Result

In light of these clinical findings, the case was diagnosed as MIRAGE syndrome. Full gene sequencing of the patient was performed. In the patient, a heterozygous one base change (c.2920G>A) leading to a missense mutation p.E974K (p.Glu974Lys) was indentified in the *SAMD9* gene. The variant was previously reported to be pathogenic with gain-of-function effect in patients with MIRAGE syndrome (2,5). This variant was not found in public SNP databases (dbSNP136, 1000 genomes, the NHLBI Exome Sequencing Project Exome Variant Server, or The Exome Aggregation Consortium). *In silico* prediction methods, SIFT and Clinvar, indicated that the mutation would be pathogenic.

Genetic analysis of parents could not be performed to confirm the *de novo* nature of the variant because the parents’ blood samples were not available. The family was requested to attend for blood sample collection, but they did not come.

Written informed consent was obtained from the patient’s family for publication of the case.

## Discussion

Adrenal hypoplasia is a rare, congenital and life-threatening disease. Patients with adrenal hypoplasia are clinically classified into two categories: the first is without any extra-adrenal features (non-syndromic adrenal hypoplasia), and the second category is with such features present (syndromic adrenal hypoplasia).

The genes responsible for the former category include those that code for the corticotropin receptor *(MCR2)* or its accessory protein *(MRAP)*, *DAX1* transcription factor *(NR0B1)*, nicotinamide nucleotide transhydrogenase *(NNT)* and mitochondrial thioredoxin reductase (TXNRD2). The syndromic category includes four different forms which are AAA syndrome (*AAAS* mutations), IMAGE syndrome (*CDKN1C* mutations), MIRAGE syndrome (*SAMD9* mutations), and a syndrome with *MCM4* mutations (2).

New advances in molecular genetics technologies have led to the identification of various rare gene defects in patients with primary adrenal insufficiency. However, 20-60% of patients with primary adrenal insufficiency remain genetically undiagnosed (6). Previous molecular genetic studies in MIRAGE syndrome have specifically targeted patients with adrenal insufficiency. The first two studies of MIRAGE syndrome were reported by studies from Japan and the United Kingdom and 94% of patients with *SAMD9* variant from these two reports had adrenal insufficiency (2,3).

MIRAGE syndrome was first described by Narumi et al (2016) (2) in 11 patients that showed strikingly similar phenotypes, including prenatal and postnatal moderate to severe growth restriction. The presence of skin hyperpigmentation, even before the onset of salt-losing symptoms in these patients, led to a suspicion of adrenal insufficiency and adrenal hypoplasia was detected via ultrasonography in seven patients. The extent of neurodevelopmental effects varied among patients as four patients out of eight, who survived the first year of life, did not have head support and any speech. Out of the seven patients with the 46,XY karyotype, underdevelopment of the genitalia with microphallus, cryptorchidism and hypospadias was observed in six; and one of the patients had complete female external genitalia at birth. During the early toddler years, all of the patients had thrombocytopenia and/or anemia that required transfusions which spontaneously resolved. Serious invasive infections, such as sepsis, meningitis and fungal infections were observed at all times; six patients died before the age of two years, mainly due to invasive infections. Two patients, who were diagnosed with chromosome 7 mosaic monosomy and developed MDS died due to complications. The MDS in these patients developed at two and three years of age. Heterozygote *SAMD9* gene mutations were detected in all of the patients (2).

The clinical features of our case were similar to previously described patients’ findings. Although mosaic monosomy 7 was detected in our patient, MDS did not develop. However, our patient died soon after presentation due to invasive infections and thus it was not possible to establish any evidence of future MDS development. The relationship between MIRAGE syndrome and MDS is complex. MDS is a heterogeneous disease, characterized by clonal hematopoiesis, the proliferation of ineffective blood cells and an increased risk of acute leukemia. In half of the patients with MDS, there are chromosomal abnormalities that most commonly include interstitial or complete deletion of chromosome 7 (7,8).

*SAMD9* is known to be a potent and widely expressed growth repressor (9,10). The first described *SAMD9* mutation responsible for human disease was the homozygote p.K1495E variant that caused familial normophosphatemic tumoral calcinosis (9). Cells with *SAMD9* mutations are characterized by structural and functional variations in the endosomal system (2). The propensity of cells to overcome the growth restriction of mutant *SAMD9* protein, somatic monosomy 7, 7q deletion or even somatic deletion-nondisjunction mutations are observed in MIRAGE patients with or without any evidence of MDS (2,3). In spite of the loss of the whole of chromosome 7, cells with loss-of-function mutations would likely gain a survival advantage over mutated cells with growth restriction. A similar “aneuploidy adaptation” mechanism in disordered cells has been reported in a mouse model with fumarylacetoacetate hydrolase deficiency (11). The first evidence of the adaptation-by-aneuploidy mechanism in humans by deletion of chromosome 7 in *SAMD9* mutation carriers was reported by Narumi et al (2016) (2).

Gonadal differentiation has a significant effect on gender development in human embryos. Understanding the developmental biology and embryology of the urogenital system is crucial to categorization and definition of the molecular basis of the disease and, if possible, the treatment of an individual patient. Sexual differentiation refers to the process through which male or female phenotype develops. Throughout the first two months of human gestation, both sexes develop in the same way. The gonads, internal genital ducts and external genital structures all develop from bipotential embryologic tissues. Each cell in the developing gonad has the potential to differentiate into either a testicular or ovarian cell.

The gonads are derived from intermediate mesoderm. In humans, between the fourth and sixth gestational weeks, the urogenital ridges develop as paired protrusions of the coelomic epithelium (mesothelium). The gonads, adrenal cortex, kidney and reproductive tract originate from the urogenital ridges. Several genes are necessary for the development of the bipotential gonad. Due to their origin as part of the developing urogenital system, ovaries and testes are initially located high in the abdomen near the kidneys. One of the earliest morphologic changes is increased proliferation and size of developing 46,XY gonads. 46,XY DSDs include disorders of testicular development, disorders of androgen synthesis and action, replacing and expanding the former category of male pseudohermaphroditism, and XY sex reversal (12).

The *SAMD9* gene is expressed in many tissues including the adrenal gland and gonad. Mutations of this gene occur with many disorders including effects in these tissues. Histological examination of placenta tissues obtained from patients with MIRAGE syndrome, performed in order to reveal DSD mechanisms, have revealed characteristic placental villous deterioration. Mutated *SAMD9* proteins have potent growth-restricting capacity, and thus they can directly cause systemic growth restriction and testicular hypoplasia. Additionally, *SAMD9* variants also affect the placenta, resulting in poor vascular supply and suboptimal human chorionic gonadotropin (HCG) stimulation. Testicular hypoplasia and insufficient HCG stimulation result in lack of testosterone synthesis. The assumption that the coexistence of the two mechanisms, the direct effect by a pathogenic variant and the indirect effect by placental insufficiency is a plausible mechanism leading to a serious clinical phenotypes (13).

Although the inheritance mode is autosomal dominant, the fact that less than 25% of all reported patients are female suggests that MIRAGE syndrome might be overlooked in girls. Female patients with 46,XX karyotype do not show any external genitalia abnormalities although ovarian dysgenesis is present histologically (2). In all of the 15 patients with 46,XY karyotype, there were external genital anomalies that ranged from hypospadias to full female phenotype (13). In addition, the early diagnosis in the current case was mainly due to the 46,XY DSD of our patient. It is possible that female patients without external genital anomalies may die before any definitive diagnosis is reached.

There is a broad spectrum of phenotype variation related to *SAMD9* (14). These variations can pose difficulties in the clinical diagnosis of MIRAGE syndrome. However, some features are notable. Adrenal insufficiency seems to be a relatively consistent feature and was the reason for the identification of the largest cohort described to date. Previous reports underline the features of the systemic disorder and, in particular, the high rates of death in MIRAGE syndrome. In order to improve the outcome, an early diagnosis that might lead to appropriate medical intervention is required. This case of MIRAGE syndrome, with a previously identified p.E974K mutation, emphasizes the dysmorphology and other findings that might assist in the earlier detection of this disorder.

## Conclusion

MIRAGE syndrome is difficult to diagnose correctly in patients with syndromic adrenal hypoplasia due to various genetic etiologies and overlapping clinical and biochemical features. This is the first report from Turkey of syndromic adrenal hypoplasia which was diagnosed with MIRAGE syndrome.

## Figures and Tables

**Table 1:**
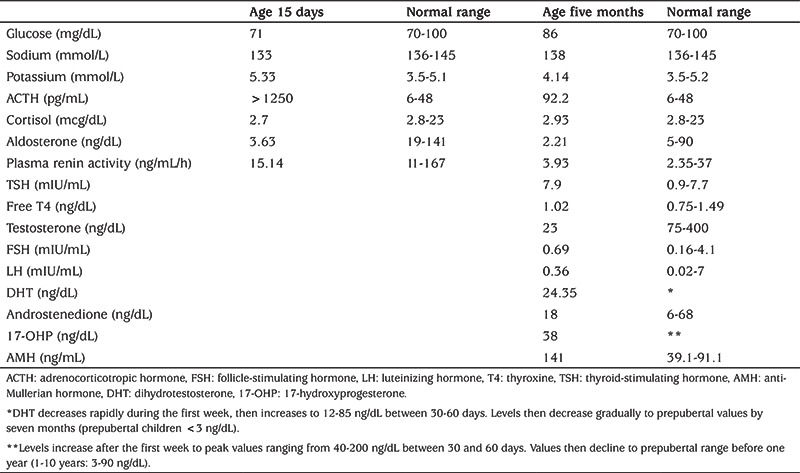
Hormonal results of the patient
